# Identification of ecdysone receptor target genes in the worker honey bee brains during foraging behavior

**DOI:** 10.1038/s41598-023-37001-7

**Published:** 2023-06-28

**Authors:** Shiori Iino, Satoyo Oya, Tetsuji Kakutani, Hiroki Kohno, Takeo Kubo

**Affiliations:** Department of Biological Sciences, Graduate School of Science, The University of Tokyo, Bunkyo-ku, Tokyo, 113-0033 Japan

**Keywords:** Animal behaviour, Molecular neuroscience

## Abstract

Ecdysone signaling plays central roles in morphogenesis and female ovarian development in holometabolous insects. In the European honey bee (*Apis mellifera* L.), however, *ecdysone receptor* (*EcR*) is expressed in the brains of adult workers, which have already undergone metamorphosis and are sterile with shrunken ovaries, during foraging behavior. Aiming at unveiling the significance of EcR signaling in the worker brain, we performed chromatin-immunoprecipitation sequencing of EcR to search for its target genes using the brains of nurse bees and foragers. The majority of the EcR targets were common between the nurse bee and forager brains and some of them were known ecdysone signaling-related genes. RNA-sequencing analysis revealed that some EcR target genes were upregulated in forager brains during foraging behavior and some were implicated in the repression of metabolic processes. Single-cell RNA-sequencing analysis revealed that *EcR* and its target genes were expressed mostly in neurons and partly in glial cells in the optic lobes of the forager brain. These findings suggest that in addition to its role during development, EcR transcriptionally represses metabolic processes during foraging behavior in the adult worker honey bee brain.

## Introduction

Transcription factors bind specific DNA sequences to activate their target genes and some are involved in their respective intracellular signaling pathways through the regulation of target genes, resulting in various physiological responses^1,2^. Ecdysone signaling is a representative signal transduction system in arthropods, in which the molting hormone, ecdysone, and the transcription factor, ecdysone receptor (EcR), induce metamorphosis^3^. In addition to morphogenesis, ecdysone signaling is involved in other biological events in insects, such as ovarian development^4^, stress responses^5^, neural remodeling^6^, courtship behavior^7^, and sleep^8^.

EcR forms a heterodimer with its co-factor ultraspiracle (USP), a vertebrate retinoid X receptor (RXR) ortholog, and acts as a transcription complex by binding unique DNA binding motifs called ecdysone response elements (EcREs) that contain 2 inverted (A/G)G(G/T)TCA at a half site with a 1-base spacer^9–11^. In canonical ecdysone signaling, 20-hydroxyecdysone (20E), an active form of ecdysone, activates the EcR-USP complex to regulate the transcription of its target early genes like *ecdysone-induced protein 75* (*E75*), *ecdysone-induced protein 93* (*E93*, known as *Mblk-1* in the honey bee), and *Broad Complex* (*BR-C*) to induce apoptosis or programmed cell death of larval tissue during pupal stages for metamorphosis^12–14^.

Despite the accumulation of knowledge about ecdysone signaling, its role and entity in the honey bee brain are largely unknown. In the European honey bee (*Apis mellifera* L.) society, female adults differentiate into 2 reproductive castes, an egg-laying queen, and sterile workers, who are engaged in various labors to maintain colony activities^15^. Ecdysone, as a reproductive hormone, functions to induce ovarian development in the queen, but not in the workers due to its low titers in the hemolymph^16,17^. On the other hand, low but significant levels of 20E have also been detected in workers^18^, and some ecdysone signaling-related genes, such as *EcR*, *E75, E74, Mblk-1,* and *BR-C* are known to be expressed in the worker brain^19–22^. In addition, *EcR* expression is induced in the worker brain by foraging behavior in not only the honey bee^23,24^ but also their close relative, the bumble bee (*Bombus ignitus*)^24^, suggesting that *EcR* has some roles associated with foraging behavior^23,24^. Whether EcR induces the expression of ecdysone signaling-related genes in the brain like during metamorphosis, however, is unknown. It is possible that EcR has acquired novel functions in the brains of workers of eusocial bees to regulate genes related to foraging behavior.

Here, we searched for *A. mellifera* EcR (*Am*EcR) target genes in the worker honey bee brain to investigate the role of *Am*EcR in foraging behavior. We prepared an anti-*Am*EcR antibody and performed chromatin immunoprecipitation sequencing (ChIP-seq) analysis using 2 types of worker bee brains: younger nurse bees taking care of the brood inside the dark hives, and older foraging bees (foragers) gathering pollen and nectar outside the hives^15^. The results suggested that *Am*EcR binds EcREs around its target genes including known ecdysone signaling-related genes and novel target genes. Most of the identified target genes overlapped in the nurse bee and forager brains, suggesting that *Am*EcR regulates common genes in the brains of nurse bees and foragers. In addition, RNA-seq analysis identified *Am*EcR target genes whose expression is upregulated by foraging behavior and single-cell RNA-seq analysis using forager brains revealed that those genes were expressed primarily in neurons and partly in glial cells. Our findings suggest that *Am*EcR upregulates not only canonical EcR target genes identified in other insect species but also novel target genes, and that *Am*EcR and ecdysone-signaling might have been adapted to regulate physiological functions such as metabolic processes in neurons in worker honey bee brains during foraging behavior.

## Results

### Preparation of the anti-*Am*EcR antibody and detection of *Am*EcR in worker honey bee tissues

**Figure 1:**
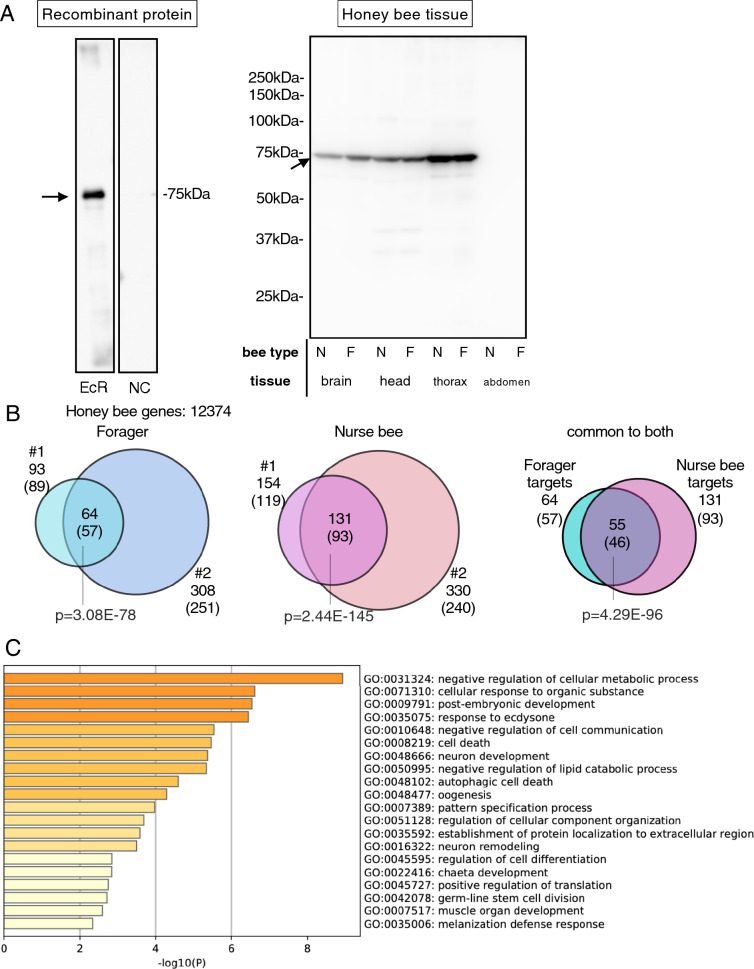
ChIP-seq analysis using *Am*EcR antibody. (**A**) Anti-*Am*EcR antibody reacted with purified *Am*EcR recombinant protein (EcR) as a single band, but not with *E. coli* lysate used as a negative control (NC). These samples were analyzed on the same membrane. The anti-*Am*EcR antibody also detected single bands in samples of each tissue from foragers (F) and nurse bees (N), except for the abdomen tissue samples. Arrows indicate the bands for *Am*EcR. (**B**) Number of *Am*EcR target genes detected in foragers (blue) and nurse bees (pink) called at p < 1E−3. #1 and #2 indicate biological replicates 1 and 2, respectively. Significance of overlaps between these ChIP-seq peaks were tested by two-side hypergeometric test using all honey bee genes (n = 12,374). (**C**) Estimation of the functions of all *Am*EcR target genes in the worker brain by GO enrichment analysis.

To search for *Am*EcR target genes by ChIP-seq analysis, we first prepared a specific antibody raised against recombinant *Am*EcR. The specificity of the prepared antibody was validated by Western blotting. An approximately 75-kDa band corresponding to one of the 2 *Am*EcR isoforms, *Am*EcR-A, was detected in the lane containing the purified recombinant full-length *Am*EcR-A, but not in the lane containing lysate of *Escherichia coli* (*E. coli*) transformed with a control vector (Fig. 1A, left panel, Supplementary Fig. S1). Single 75-kDa bands were also detected in some of the worker tissues: brains, heads without brains, and thoraxes, but not in the abdomen (Fig. 1A, right panel), indicating that this antibody specifically recognizes *Am*EcR and that *AmEcR*-A is predominantly expressed in various honey bee tissues. The results also suggested that *Am*EcR functions in both nurse bees and foragers.

### Identification of *Am*EcR target genes in the nurse bee and forager brains using ChIP-seq analysis

We then conducted ChIP-seq analysis using the anti-*Am*EcR antibody to search for *Am*EcR target genes in the honey bee brain.

DNA was recovered from the chromatin solutions extracted from the nurse bee and forager brains by immunoprecipitation using either the anti-*Am*EcR antibody or rabbit-normal IgG as a negative control. The summary of sequences is shown in Supplementary Table S1. By comparing the mapped reads of anti-*Am*EcR samples with those of negative control using MACS2^25^, 93 and 308, and 154 and 330 *Am*EcR binding sites were detected in each of replicate #1 and #2 of the forager and nurse bee brain samples, respectively, and 89 and 251, and 119 and 240 genes closest to those sites were identified as candidate *Am*EcR target genes (Fig. 1B and Supplementary Table S2). Peaks that were reproducibly detected between 2 biological replicates were extracted as reliable candidate targets: 64 peaks (57 genes) and 131 peaks (93 genes) were identified in the nurse bee and forager brain, respectively (Fig. 1B), indicating that significant portions of the genes were reproducibly detected in the replicates of both forager and nurse bee (hypergeometric test, forager common genes: p = 3.08E−78, nurse bee common genes: p = 2.44E−145). A comparison of the peaks common to the forager and nurse bees showed that the majority of the *Am*EcR peaks (55) were detected in both bees (Fig. 1B, hypergeometric test, p = 4.29E−96, Supplementary Fig. S2A). The DNA sequences located near the detected peaks were also almost the same between the nurse bee and forager, although a small number of the target peaks were specific to either nurse bee or forager (Supplementary Fig. S2B, div3 and div5). The 46 common target genes included some ecdysone signaling-related genes, such as *hormone receptor 4* (*Hr4*), *E75*, and *kruppel homolog 1* (*Kr-h1*), reported in other insect species^26,27^. In contrast, some of the other known EcR targets, such as *ftz transcription factor 1* (*Ftz-f1*)*, BR-C*, and *E93*^28,29^*,* were not detected as candidate *Am*EcR target genes in either the nurse bee or forager. This finding suggests that some genes are continuously targeted by *Am*EcR from pupal to adult stages, whereas others are targeted only during the pupal stages.

To estimate the possible function of *Am*EcR in the worker brain, GO enrichment analysis was conducted using 104 genes identified as at least 1 of the target genes in the nurse bee or forager. As a result, the identified GO terms included some biological processes induced by ecdysone signaling, such as "post-embryogenic development" (ranked 3rd), "response to ecdysone" (ranked 4th), "autophagic cell death" (ranked 9th), “oogenesis” (ranked 10th), as well as neuron-specific functions like “neuron development” (ranked 7th) and “neuron remodeling” (ranked 14th) (Fig. 1C, Supplementary Table S3). It also included negative regulation of cellular metabolic processes (ranked 1st), that of cell communication (ranked 5th), and lipid catalytic processes (ranked 8th), suggesting a novel role of *Am*EcR in repressing metabolism in the worker brain.

### *Am*EcR also binds EcREs in adult worker brains

**Figure 2:**
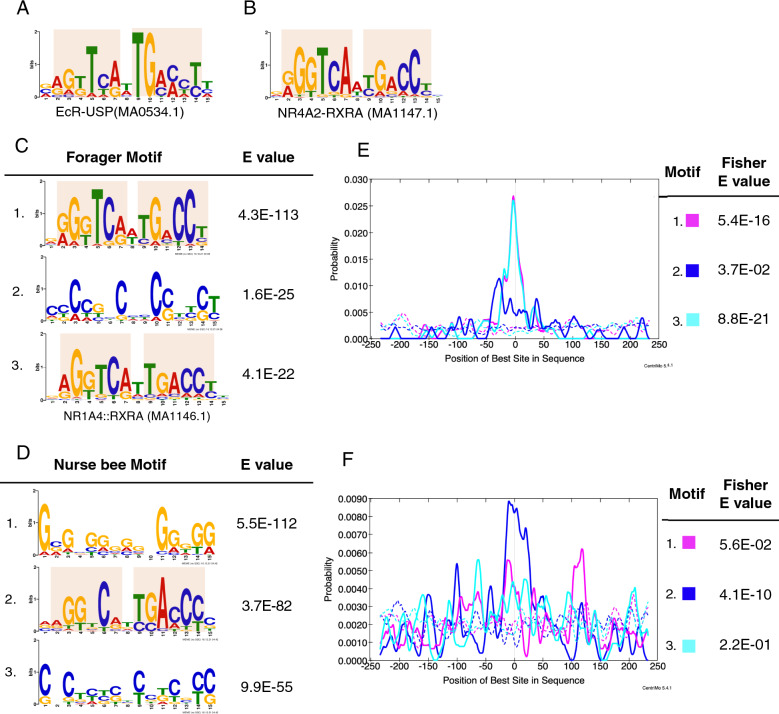
Motif analysis. (**A**,**B**) The most similar motif detected in Tomtom was the motif of EcR-USP (registered name: MA0534.1) (**A**) and that of NR4A2-RXRA (registered name: MA1147.1) (**B**) in the database JASPAR 2018. (**C**,**D**) Top 3 motifs detected in MEME-ChIP discriminative mode from *Am*EcR-ChIP binding sequences of foragers (**C**) and those of nurse bees (**D**). E values are shown beside each motif. Conserved EcR-USP half motifs are indicated by shading. (**E**,**F**) Localization of motifs detected in **C** (**E**) and **D** (**F**) using CentriMo. Enrichment of each detected motif in the ChIP binding sequences is indicated by different colored lines. Dotted lines indicate localization of the motifs in the sequences randomly extracted from the honey bee genome set as negative controls. Fisher E values are shown beside each motif.

EcR and USP form a heterodimeric complex to bind EcREs around the target genes (Fig. 2A)^9–11^. We examined whether *Am*EcR also binds EcREs even in the adult brain. First, we searched for the DNA motifs that are enriched in the DNA sequences ± 250 bases from the peak summits, using MEME-ChIP. The top hit motif detected in the forager sequences was a palindrome-like motif (Fig. 2C). A similar palindrome-like motif was also detected as the second hit in the nurse bee sequences, although the G-rich motif was estimated as the top (Fig. 2D). Further examination of the localization of each motif in the same sequences (± 250 bases from the peak summit center) indicated that both palindrome-like motifs were significantly enriched at the center of the sequences (Fig. 2E, Fisher E-value compared with negative control sequences E = 5.4E−16; Fig. 2F, Fisher E = 4.1E−10). In contrast, the G-rich motif, the top motif detected in the nurse bee sequences, was not significantly enriched at the peak center (Fig. 2F, Fisher E = 5.6E−2). These observations suggested that the palindrome-like motifs are most likely *Am*EcR binding sequences in both nurse bee and forager brains. When motifs similar to these motifs were searched for using Tomtom software, both palindrome-like motifs were most similar to that of NR4A2-RXRA, a transcription factor complex of the nuclear receptor subfamily and mammalian USP ortholog RXR (Fig. 2B; similarity to motif 1 in Fig. 2C, p = 9.3E−13; similarity to motif 2 in Fig. 2D, p = 1.5E−14). They were also similar to the *Drosophila* EcR-USP motif (Fig. 2A; similarity to motif 1 in Fig. 2C, p = 1.3E−8; the similarity to motif 2 in Fig. 2D, p = 8.2E−8). These results indicated that, like during metamorphosis, *Am*EcR recognizes EcREs in both nurse bee and forager brains.

### Expression analysis of *Am*EcR target genes in worker brains before/after foraging behavior

**Figure 3:**
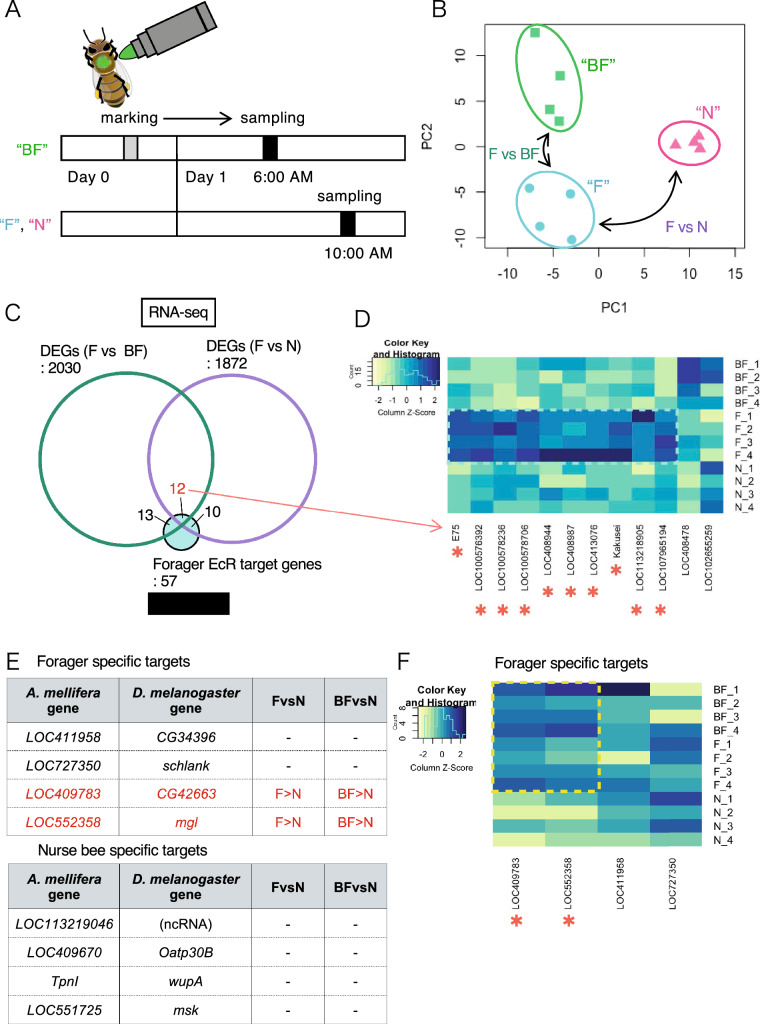
Expression levels compared between before/after foraging and between the division of labor. (**A**) Overview of sampling timescales for RNA-seq. A gray bar indicates marking time and black bars indicate the sampling times. F: forager during foraging behavior, N: nurse bee in the hive, BF: forager before foraging. (**B**) PCA plot of RNA-seq samples. (**C**) Numbers of DEGs between foragers and foragers before foraging (green) and those between foragers and nurse bees (purple) detected in RNA-seq and numbers of *Am*EcR target genes in foragers detected by ChIP-seq (light blue). DEGs: differentially expressed genes. (**D**) Gene expression levels of 12 target genes classified in (**C**). (**E**,**F**) Expression levels of 4 forager-specific targets and 4 nurse bee-specific targets. The tables show target genes, their homologs in *D. melanogaster*, determination of DEGs between forager and nurse bee, and those between foragers before foraging and nurse bees.

As *AmEcR* is upregulated by foraging behavior^23,24^, we hypothesized that some *Am*EcR target genes are also transcriptionally regulated in response to foraging behavior. Therefore, we used RNA-seq analysis to investigate whether the expression levels of the 57 target genes identified by ChIP-seq in the forager brain change according to foraging behavior. We also compared the expression levels of target genes in the nurse bee and forager brains to examine whether they are differentially regulated depending on the age and/or division of labor from nurse bees to foragers. For this, we collected 3 groups of workers: nurse bees (N), foragers before foraging (BF), and foragers after foraging (F), as follows (Fig. 3A). N and F were collected at 10:00 AM. As BF, we marked the foragers with pollen loads that returned to the hives after foraging on the previous day (Day 0) and collected the marked individuals from the hives at 6:00 AM the next morning (Day 1) before they left for foraging. RNA-seq was performed using 4 lots each of the whole brain samples of these 3 groups (BF, F, and N) to detect differentially expressed genes (DEGs) among those groups (Fig. 3B, Supplementary Fig. S3A and Supplementary Table S4).

A total of 2030 and 1872 genes were detected as DEGs between BF and F, and F and N, respectively. Among the 57 *Am*EcR target genes identified in the forager brain by ChIP-seq analysis, 25 and 22 were identified as DEGs in each set (F vs. BF, and F vs. N, respectively), and 12 were common DEGs to both sets (F vs. BF and F vs. N; Fig. 3C; shown in red in Supplementary Table S5). Among these 12 DEGs, the expression levels of 10 genes: *E75*, *kakusei, LOC100576392*, *LOC100578236*, *LOC100578706, LOC113218905*, *LOC408944*, *LOC408987, LOC413076*, and *LOC107965194* were higher in F than in either BF or N (Fig. 3D, asterisks), suggesting that expression of these 10 genes in the brain was enhanced by foraging behavior, possibly downstream of the late-upregulated *Am*EcR^23,24^. These 10 genes included ecdysone signaling-related genes such as *Eip75* (*E75*)^28^, *ecdysone-induced protein 78C* (*Eip78C, LOC413076*)^30^, and *Myc* (*LOC100576392*), which are associated with organ or cell growth^31,32^, and *musashi* (*msi, LOC100578236*), which encodes an RNA-binding protein and is associated with neural generation processes^33,34^. The 10 genes also included those not directly related to ecdysone signaling, such as *RING-associated factor 2* (*RAF2, LOC100578706*), which encodes a polycomb protein that binds RNA polymerase II^35^; *JmjC domain-containing histone demethylase 2* (*JHDM2, LOC408944*), which encodes an H3K9 demethylase enzyme^36^; *sortilin related receptor 1* (*SORL1, LOC408987*), which encodes a cellular transporter protein^37^; and some non-coding RNAs (*kakusei, LOC113218905,* and *LOC107965194*). GO enrichment analysis of these 10 genes revealed "negative regulation of macromolecule biosynthetic process" as the second GO terms for biological processes and "transcription regulation activation" as the top GO term for molecular function (Supplementary Fig. S3B, Supplementary Table S6). Of the remaining 2 DEGs among the common 12 DEGs, the expression of *LOC102655259* (*l(2)10685*) was lower in F than in BF and N, and the expression of *LOC408478* (*Tret1-2*) was higher in F than in N, but lower than in BF (Fig. 3D), which indicates that only the former gene is downregulated by *Am*EcR during foraging behavior, but its function is unknown.

In contrast, 13 genes were identified as DEGs in F vs. BF, but not in F vs. N (Fig. 3C). Among these 13 genes, 11 were expressed at higher levels in F than in BF: *InR-2* (ortholog of *D. melanogaster InR*), *LOC408298* (*pum*), *LOC409961* (*Hrb27C*), *LOC412746* (*CABIN1*, ortholog in *Homo sapiens*), *LOC552519* (*clu*), *LOC724535* (*Smr*), *LOC724704* (*orb2*), *LOC100578165* (there is no ortholog corresponding to this gene), and non-coding RNAs: *LOC1005777237*, *LOC100578461*, and *LOC113218619*, whereas the remaining 2 genes were expressed at lower levels in F than in BF: *LOC552028* (*F13H10.3*, *Caenorhabditis elegans*) and *LOC412863* (*Utx*) (Supplementary Table S5). We consider that expression changes of these 13 genes before/after foraging may reflect a difference in the sampling time (6:00 AM and 10:00 AM) rather than foraging behavior because their expression levels were almost the same between F and N, both of which were collected at 10:00 AM. GO enrichment analysis also indicated negative regulation of signaling (Supplementary Fig. S3C and Supplementary Table S6B).

### Expression analysis of nurse bee-/forager-specific *Am*EcR target genes

We then focused on *Am*EcR target peaks detected by ChIP-seq in either a nurse bee or forager brain-specific manner to investigate whether they are related to the differential physiological states between nurse bees and foragers rather than foraging behavior. As 9 peaks of "forager targets", which were specified by subtracting the 55 peaks of "common to both" from the 64 peaks of "forager targets" in Fig. 1B contained peaks detected in either of the nurse bee replicates, we newly obtained 4 "forager-specific peaks", corresponding to 4 genes, by removing those peaks (Supplementary Fig. S2B, div3). Similarly, by subtracting peaks detected in either or both of the forager replicates, from 131 peaks of "nurse bee targets", we newly obtained 11 "nurse bee-specific peaks", corresponding to 4 genes (Supplementary Fig. S2B, div5). Among the 4 genes identified as forager-specific target genes (genes labeled "Yes" in the "Forager specific?" column in Supplementary Table S5), 2 genes (*LOC409783* and *LOC552358*) were expressed at higher levels in foragers (F and BF) than in nurse bees (N) (Fig. 3E,F). It is possible that expression of these genes is upregulated in foragers by *Am*EcR in association with the age and/or physiological state of workers as well as foraging behavior. No significant change in gene expression levels was detected for the remaining 2 genes (*LOC411958* and *LOC727350*), so the role of *Am*EcR in the regulation of these 2 genes on the division of labor is unclear.

Similarly, although 4 genes were identified as nurse bee-specific target genes (3 nurse bee-specific peaks including 1 located at an overlapping region of *LOC409670* and *LOC113219046* on the honey bee genome), none of them were differentially expressed between forager and nurse bees (Fig. 3E, labeled "Yes" in the "Nurse bee-specific" column in Supplementary Table S7), and thus the role of *Am*EcR in the regulation of these 4 genes in association with the division of labor is unclear.

### Cell type-specific expression of *Am*EcR and its target genes in the forager brain

**Figure 4:**
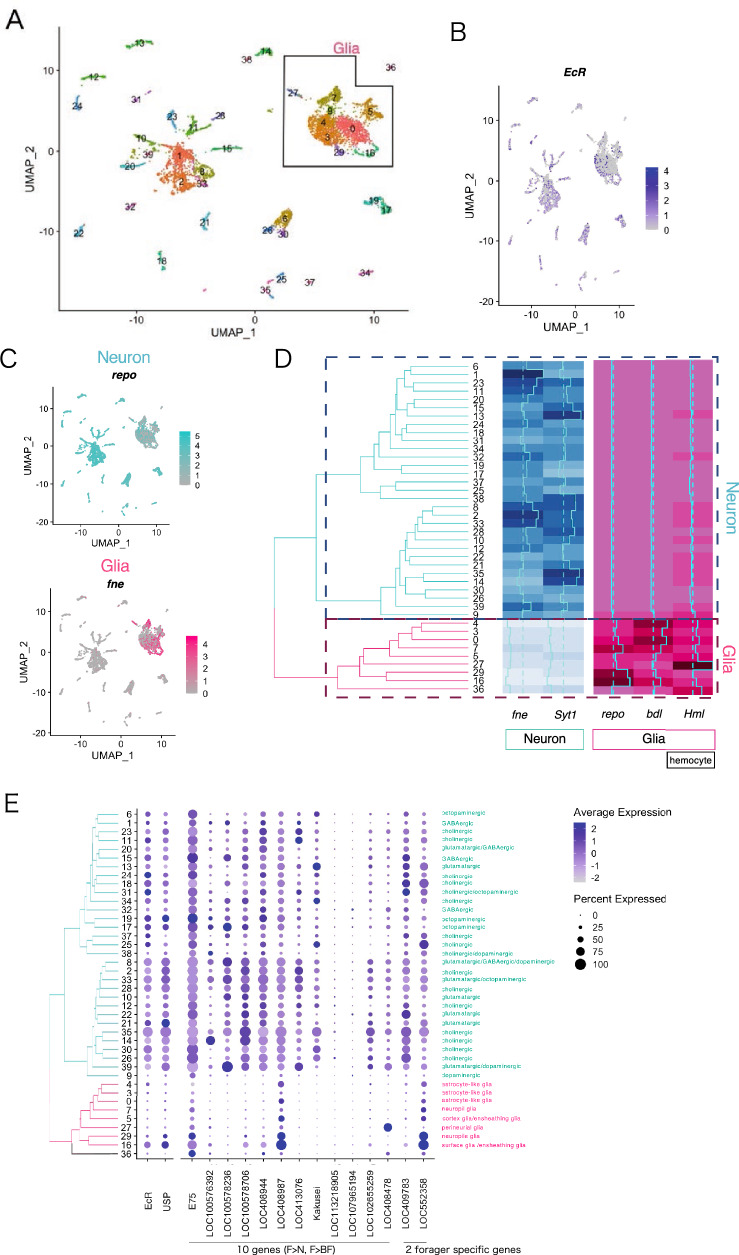
Cell types expressing *AmEcR* and its target genes in the forager brain. (**A**) UMAP plot of single-cell RNA-seq. Clusters of glial cells are enclosed by a black line. (**B**,**C**) Expression of *EcR* (**B**), neuron marker *repo* and glial marker *fne* (**C**) in (**A**). (**D**) The hierarchical clustering tree of UMAP clusters (left) and the expression levels of marker genes of neurons (blue) and of glial cells (magenta) in each cluster based on pseudo bulk differential expression analysis (right). (**E**) Dot plot indicates expression levels and percentage of cells expressing *EcR*, its co-factor *USP*, and its 12 target genes; 10 genes are the DEGs upregulated in foragers indicated by asterisks in Fig. 3D, and 2 genes are forager-specific genes indicated in Fig. 3F.

Because both "negative regulation of macromolecule biosynthetic process" and "transcription regulation activation" were identified as major GO terms for the molecular functions of the *Am*EcR target genes (Supplementary Fig. S3B, Supplementary Table S6) and metabolism in animal brains is generally more active in glial cells than in neurons^38^, we assumed that these genes might be induced in glial cells in the forager brains. Previous in situ hybridization analysis suggested that *AmEcR* is strongly expressed in a subset of neurons termed Kenyon cells in the mushroom bodies (MBs), a higher-order center in the insect brain^20^, but recent real-time PCR analysis revealed that *AmEcR* expression levels are higher in other brain regions, including the optic lobes (OLs), a visual center, which occupy a large portion of the honeybee brain^24^. Therefore, *AmEcR* and its target genes may be expressed in glial cells rather than neurons in the OLs. Given that previous single-cell RNA-seq (scRNA-seq) analyses of *Drosophila* OLs clearly classified glial and OL neuron types^39,40^, and that the cell types of the OLs are more conserved between *Drosophila* and Aculeata (ant) than those of the MBs^41^, we performed scRNA-seq of the OLs of foragers using a 10 × genomics chromium platform to determine whether *AmEcR* and its target genes are expressed in the glia or neurons in the OLs. By subsequent data analysis using the Seurat pipeline, we obtained 6843 cells with a minimum of 2000 unique feature count (mapped to a total 10,861 genes), which were divided into 40 clusters (Fig. 4A). We branched these 40 clusters into 2 main clusters, neurons and glial cells, by hierarchical clustering and the expression levels of the neuron markers (*fne* and *Syt1*) and the glial cell markers (*repo* and *bdl*) in each cluster^39,40^ (Fig. 4C,D; Supplementary Fig. S4A). Among the 40 clusters, cluster 36 had the second highest expression of *Hml*, the hemocyte marker gene, and the correlation between the cluster 36 and each cluster was low, suggesting that the cells in cluster 36 contain hemocytes (Supplementary Fig. S4B). Consistent with a previous report^39^, expression of the projection neuron marker separated cholinergic neurons and glutamatergic neurons regardless of the branching based on hierarchical clustering (Supplementary Fig. S4C). Based on these classifications, *AmEcR* was expressed in various neural clusters, with especially strong expressions in some clusters, such as clusters 17, 19, 24, and 31, which corresponded to cholinergic or octopaminergic neurons, respectively (Fig. 4B,E). In addition, moderate expression was also detected in a restricted glial cell cluster, cluster 16, which corresponded to both surface glia and ensheathing glia.

Among 12 *Am*EcR target genes, such as *E75, LOC100576392, LOC100578236,* and *LOC413076*, were mainly expressed in various neurons in which the *AmEcR* expression was detected (Fig. 4E). In contrast, although expression of 2 genes, *LOC408987* and *LOC552358*, both of which encode lipoproteins, was detected in both various neurons and some glial cell clusters, such as clusters 16 and 29, where *AmEcR* was also expressed, their expression was stronger in glial cell clusters than in neural clusters (Fig. 4E). These results strongly suggested that *Am*EcR regulates different target genes in neurons and glial cells.

## Discussion

The findings of the present study demonstrated that not only some genes participating in canonical ecdysone signaling related to metamorphosis and ovary development but also novel genes that have not been detected as EcR targets in previous research^42–45^ are induced by *Am*EcR in the worker honey bee brain during foraging behavior, and suggested that they function to repress metabolic processes.

Our Western blotting experiments using anti*-Am*EcR antibody, which recognizes a common region of 2 *Am*EcR isoforms, detected *Am*EcR as a single band of approximately 75 kDa in honey bee tissues (Fig. 1A). Considering that the calculated molecular weights of *Am*EcR-A and *Am*EcR-B are 66.6 kDa and 60.7 kDa, respectively, it is likely that mainly *Am*EcR-A is expressed in various honey bee tissues. *Am*EcR was not detected in the worker abdomens (Fig. 1), which is consistent with previously reported Northern blotting results^20^, possibly because *Am*EcR does not function in the worker abdomen, which has shrunken ovaries.

ChIP-seq analysis using the anti-*Am*EcR antibody identified some known EcR target genes and functions (Fig. 1C and Supplementary Table S5), which were identified in previous studies using other insect species and cultured cells^42–45^. In addition, GO enrichment analysis indicated that *Am*EcR regulates genes involved in known ecdysone signaling functions (Fig. 1C). On the other hand, some known EcR target genes, such as *E93*, *BR-C*, and *Ftz-f1,* were not detected in the present study, although these genes are expressed in the adult honey bee brain^19,22,46^. Considering that *Ftz-f1* and *BR-C* are involved in switching the division of labor of workers independently from ecdysone signaling^47^, it is possible that these genes are no longer *Am*EcR targets in the adult worker brain and have their own expression mechanisms.

The binding motif analysis identified EcREs as binding motifs of *Am*EcR even in adult worker brains (Fig. 2). Although other proteins involved in the juvenile hormone response, such as Met, FKBP39, and Chd64, also form a complex with EcR in *Drosophila*^48^, neither the Met-USP motif^49^ nor the juvenile hormone response element which is bound by FKBP39 and Chd64^48^ was detected as a binding motif for *Am*EcR in the present study. These results suggest that *Am*EcR forms a heterodimeric complex with USP to regulate the transcription of target genes in the worker brain, which is consistent with previous reports that some *Am*EcR target genes are consistently expressed in adult worker brains^19–22,46^. Besides EcREs, the G-rich or C-rich motifs detected in the motif search (Fig. 2C,D) were shown using the motif estimation tool to resemble the binding elements of C_2_H_2_ zinc finger transcription factors, such as *Znf281*, *Znf263*, and *SP2*. It is possible that these other transcription factors bind near the binding sequence of *Am*EcR and together contribute to the transcriptional regulation of target genes.

RNA-seq analysis revealed that 10 *Am*EcR target genes in the forager brain were upregulated in association with foraging flight (Fig. 3D, asterisks). In the present study, we sampled ‘nurse bees’ and ‘foragers’ based on their behavior as well as the morphology of their hypopharyngeal glands, which are well developed in nurse bees but shrunken in foragers, as reported previously^50^, and not on their age after eclosion, because we aimed to identify genes that are transiently induced in the brain depending on foraging behavior rather than merely on their physiology or foraging experience. The variation between biological replicates was rather large, which might be partly due to the differences in the physiology and/or foraging experience of the workers used for the analysis. The GO enrichment analysis suggested that most of these genes, such as *E75*, *E78C*, and *msi*, are involved in "negative regulation of macromolecule biosynthetic processes" (Supplementary Table S6A). In *Drosophila*, *E75* is involved in lipid accumulation in germ cells, and the mammalian ortholog of *E75*, *Rev-Erb*, regulates genes involved in lipid metabolism^51,52^. *E78C* regulates triacylglycerol lipase and is involved in maintaining adult lipid levels in *Drosophila*^53^. Besides the above genes, *JHDM2* functions to regulate genes related to energy metabolism in mice, as its mutant becomes obese^54^. *SORL1* (*sortilin-related recepto*r) has never been described in the context of ecdysone signaling, although it codes a low-density lipoprotein that functions in intracellular vesicle trafficking and lipid transportation, and is involved in metabolic regulation^55^. A recent study in *Drosophila* showed that ecdysone signaling is involved in the regulation of metabolic system to reduce energy consumption during post-pupal starvation^56^ and in lipid accumulation during female ovarian development^52^. Based on these previous findings, *Am*EcR targets in the worker brain might be related to lipid metabolism.

Honey bee foragers have less lipid storage in their abdominal fat bodies than nurse bees^57^, and the expression levels of genes involved in lipid metabolism are also lower in the fat bodies of foragers than in those of nurse bees^58^. Nonetheless, it is possible that foragers require a lot of energy during foraging behavior, not only for contraction of flight muscles^59^ but also for neural activity in the brain associated with foraging behavior^60,61^, and therefore need to suppress unnecessary energy consumption in neurons or glial cells in the brain^60,62^. On the other hand, in *Drosophila*, ecdysone signaling induces lipolysis during the larva to pupa transition, where EcR and some target genes, such as *E75* and *Myc*, are involved in suppressing lipid storage in the fat body^63,64^. Therefore, another possibility is that the honey bee brain obtains energy from lipolysis by *Am*EcR during foraging behavior.

Another characteristic of the *Am*EcR target genes is that some of them: *E75*, *JHDM2*, *Myc*, and *EcR* are regulated by circadian rhythms in *Drosophila*^9,65–67^. In the honey bee, expression of *EcR* and some ecdysone signaling-related genes, such as *E75*, oscillates according to the circadian rhythms in the forager brain but not in the nurse bee brain^68,69^. Thus, it is possible that the induction of some *Am*EcR target genes in the brain is controlled by daily rhythms and that foragers prepare for changes in nutritional status associated with foraging flight by regulating metabolism through the *Am*EcR-mediated signaling system.

The *Am*EcR target genes also included several non-coding RNAs, such as *kakusei*^70^. *kakusei* is an immediate early gene unique to the honey bee which is induced by foraging behavior in the brain^70^, and its expression changes in response to food availability^23^. Thus, it might be that after the immediate induction, the expression level of *kakusei* is tuned in response to food-related information under the control of late-induced *Am*EcR. It is noteworthy that some non-coding RNAs (*kakusei*, *LOC113218905*, and *LOC107965194*), *SORL1*, *JHDM2*, and *RAF2*, which are not known to be direct targets of EcR, were identified as *Am*EcR target genes, raising a possibility that *Am*EcR acquires novel functions in the worker honey bee brain beyond the known functions of ecdysone signaling. The actual functions of most of the target genes mentioned above, however, have not been investigated in the brains of honey bees or other animal species, and further research is needed to reveal the functions of those genes.

ChIP-seq analysis also identified target genes corresponding to 4 forager-specific peaks and 3 nurse bee-specific peaks (Fig. 3E). Among the 4 genes corresponding to forager-specific peaks, 2 genes, the orthologs of *CG42663* and *megalin* (*mgl*), were upregulated in the forager brain compared with the nurse bee brain. *CG42663* is involved in lifespan in *Drosophila*^71^, and it is possible that the regulatory system of this gene changes in association with the age-dependent division of labor. *mgl* is a member of the low-density lipoprotein receptor family and mice lacking *mgl* in the brain endothelial cells exhibit obesity^72^. *mgl* is also involved in memory and learning mechanisms by increasing mature spines and active synapses in mice^73^. Thus, the promotion of *mgl* expression by *Am*EcR in the forager brain may be related to the low-density lipoprotein metabolism and/or the increased dendritic branching observed in the MBs of forager brains^74^.

Finally, single-cell RNA-seq analysis revealed that *Am*EcR and its target genes upregulated in the forager brain, such as *E75*, *LOC413076* (*Eip78C*), *LOC100576392* (*Myc*), *LOC408944* (*JHDM2*), and *LOC100578706* (*RAF2*), are mainly expressed in various types of neurons rather than glial cells in the optic lobes (Fig. 4E). It is possible that ecdysone-signaling is involved in the metabolism in various OL neurons of honey bees during foraging behavior, although these findings are somewhat opposed to the previous notion that glial cells rather than neurons are generally engaged in lipid metabolism^38^. These results also seem to be consistent with previous report that visual information is important for the foraging flight of the honey bee^75^. In contrast, 2 lipoprotein genes, *LOC408987* (*SORL1*) and *LOC552358* (*mgl*), were more highly expressed in glial cells (Fig. 4E), suggesting their roles in lipid transport in the worker brain during foraging behavior. These target genes were expressed in ensheathing glia, which functions as a neuronal insulator^76^, and surface glia, which forms the blood–brain barrier^77^. Especially, surface glia responds by accepting hormones from the bloodstream. Surface glia are also involved in glucose metabolism required for neuron survival^78^ and *SORL1*, which is highly expressed here, enhances insulin receptivity^79^, suggesting that *SORL1* promotes induction of the insulin signaling pathway in glial cells. Contrary to our initial expectations, some of the *Am*EcR targets were expressed in neurons, suggesting the importance of investigating the cell types that express *Am*EcR target genes in brain regions other than the OLs, such as the MBs, central complex, antennal lobes, and suboesophageal ganglion, in future studies.

In the present study, we propose a novel role for *Am*EcR expressed in the brain of worker bees during foraging behavior. To evaluate those hypotheses empirically, further studies are needed to examine the functions of *Am*EcR by pharmacological inhibition or knockdown of the target genes using RNA interference experiments^80^.

## Materials and methods

### Bees

European honey bee colonies were purchased from Kumagaya Beekeeping Co. Ltd. and Rengedo Inc., and maintained in the University of Tokyo. Nurse bees and foragers were caught on the basis of their behaviors and the development of hypopharyngeal glands, which are well-developed to secrete royal jelly in nurse bees and shrunken in foragers^80^. Sampling for the ChIP-seq analysis was performed in February and November 2021 for the foragers, and in April and November 2021 for the nurse bees for two biological replicates. Sampling for RNA-seq analysis was performed in July 2021. Foragers with pollen loads on their legs were marked on the day before the sampling day, and caught inside the bee hive at 6:00 AM the next morning as “before foraging” (BF). Foragers and nurse bees were collected at 9:30–10:00 AM on the same day. Sampling of foragers for single-cell RNA-seq was performed in October 2020.

### Anti-*Am*EcR antibody preparation

Full-length *Am*EcR-A (NCBI accession number: NM_001098215.2) was cloned into a pCold I vector (Takara), which is used for protein expression at low temperatures. The constructed vector was transformed into *E. coli* (BL21 [DE3]), and the expression of *Am*EcR recombinant protein with a 6 × His tag at the N-terminus was induced by adding cold shock and isopropyl-β-d-thiogalactopyranoside (final conc. 1 mM). Recombinant *Am*EcR lysed in phosphate-buffered saline (PBS) and sonicated was purified using Ni-NTA His-Bind Superflow Resin (Novagen). The purified *Am*EcR was subjected to sodium dodecyl sulfate (SDS)-polyacrylamide gel electrophoresis followed by Coomassie brilliant blue staining, and the gel portion that corresponds to the band for the recombinant protein was excised and sent to Kiwa Laboratory Animals Co., Ltd for antigen sensitization in rabbits. Anti-*Am*EcR antibodies were affinity-purified from the resulting rabbit antisera using the peptide fragment (NM_001098215.2, + 744 to + 1952) that corresponds to the sequence common to 2 *Am*EcR splicing variants, *Am*EcR-A and *Am*EcR-B1 (NM_001159355.1), which was prepared by the same methods as the recombinant *Am*EcR described above. The reaction of anti-*Am*EcR antibody to its antigen was checked by enzyme-linked immunosorbent assay (Eurofins).

### Western blotting

For detection of the recombinant *Am*EcR protein, purified recombinant *Am*EcR that was used for the immunization and lysate of *E. coli* transformed with pCold I vector containing the *Am*HR38 gene fragment as negative control were used. For the detection of intrinsic *Am*EcR, bee lysate was prepared by cutting each tissue (10 brains, 3 heads without brains, 2 thoraxes, and 2 abdomens) into small pieces with scissors, and homogenizing them in SDS lysis buffer (5 mM Tris–HCl, pH 6.8, containing 2% SDS and 10% glycerol). The concentration of each lysate was measured by BCA protein assay (Pierce), and the same amount of protein for each sample was subjected to SDS-polyacrylamide gel electrophoresis and electrotransferred onto polyvinylidene difluoride membranes. Western blotting was performed essentially as described previously^81^; the blotted membrane was soaked in blocking buffer (5% skim milk in Tris-buffered saline; TBS-T [25 mM Tris–HCl, pH 7.4, 137 mM NaCl, 2.7 mM KCl, containing 0.4% Tween-20]) for 1 h, and incubated with anti-*Am*EcR purified antibody in blocking buffer overnight at 4 °C on a shaker. After washing 3 times in TBS-T on the shaker, the membranes were incubated with the secondary antibody (Goat Anti-Rabbit IgG H + L Antibody, Peroxidase-Labeled, KPL Inc.) for 1 h. After another 3 washes in TBS-T, chemical luminescence was detected using ECL Select Western Blotting Detection Reagent and Image Quant LAS 500 (GE Healthcare).

### ChIP-seq analysis

A total of 120 worker brains were used in a set for ChIP-seq analysis using anti-*Am*EcR antibody and rabbit normal IgG (Wako) as a negative control. The brains were homogenized using pestles in buffered insect saline (20 mM Tris–HCl pH 7.4, 130 mM NaCl, 5 mM KCl, 1 mM CaCl_2_, 20 mM HEPES, containing protease inhibitor cocktail). The DNA and associated proteins were crosslinked by adding 1% formaldehyde and incubating at room temperature for 12 min, and then the reaction was stopped by adding 125 mM glycine solution. The samples were centrifuged at 3300 rpm and the precipitate was washed three times in buffered insect saline and homogenized using plastic beads and Micro Smash MS-100 (Tomy) at 5000 rpm for 25 sec. After centrifugation at 5700 rpm for 10 min, the pellet was resuspended in lysis solution buffer (50 mM Tris–HCl, pH 7.4, 10% glycerol, 0.5% Nonidet P-40 substitute, and 2 mM ethylenediaminetetraacetic acid [EDTA]) and kept on ice for 30 min. After centrifugation, the pellet sample was resuspended in micrococcal nuclease (MNase) buffer (50 mM Tris–HCl, pH 7.5, 5 mM CaCl_2_) for 15 min on ice, centrifuged, and the pellet was resuspended in MNase buffer with MNase (NEB) for enzyme digestion for 10 min at 37 °C, and the reaction was stopped by adding 33 mM EDTA solution. ChIP dilution buffer (1.5-fold of the sample volume; 50 mM Tris–HCl, pH 7.5, containing 0.167 mM NaCl, 1.1% Triton-X 100, and 0.11% sodium deoxycholate) was added to the sample and the mixture was sonicated using Sonifier 450 (Branson). After the sample was centrifuged at 12,000 rpm, the supernatant was collected. Immunoprecipitation was performed with Dynabeads protein A (Thermo Fisher) according to the manufacturer’s protocol. The beads (50 μl) were washed and resuspended in PBS (pH 7.4) containing 0.02% Tween 20, and incubated for 10 min with 10 μg of each antibody: anti-*Am*EcR antibody and rabbit normal IgG. The supernatant sample for immunoprecipitation was mixed with the bead-antibody complex solution and incubated overnight at 4 °C with rotation. After washing the beads in PBS (pH 7.4) containing 0.02% Tween20, the protein-DNA complex was eluted from the beads in the elution buffer (50 mM Tris–HCl pH 7.5, 8.5 mM EDTA, and 1% SDS). The DNA sample was treated with RNase A for 1 h and then with proteinase K overnight at 50 °C, purified using phenol–chloroform followed by ethanol precipitation, and dissolved in Tris–EDTA buffer (10 mM Tris–HCl, pH 7.5, 1 mM EDTA). DNA libraries were subsequently prepared using KAPA HyperPrep Kits (NIPPON Genetics) and 150 bp pair-end sequenced on a Hiseq X (Illumina).

### RNA-seq analysis

4 lots (3 brains per lot) for each bee sample (i.e., nurse bees, foragers, and “BF” foragers) were subjected to RNA-seq analysis. The bee brains were homogenized with pestles in TRIzol Reagent (Thermo Fisher), and the RNA was extracted using a Direct-zol RNA kit (ZYMO research). After adjusting the RNA concentration to 100 ng/µl and library preparation, the samples were sequenced with MGI tech DNBSEQ G-400 (Azenta).

### Single-cell RNA-seq analysis

Optic lobes of 6 foragers were dissected and the retinas were trimmed using a dissecting scalpel. The tissues were torn and shaken for 5 min at 37 °C in enzyme solution (1 mg/ml papain, 1 mg/ml collagenase, in Dulbecco’s PBS) and pipetted 250 times until the large mass of tissue was not visible. The sample was washed twice, resuspended in Dulbecco’s PBS containing 4% bovine serum albumin and filtered with 55-μm opening nylon mesh (3-3069-10, AS ONE). The live cells were counted in a dye exclusion test using 0.4% trypan blue solution and a hemocytometer. The sample was on the pipeline of 10 × Genomics Chromium single-cell RNA-seq (Azenta). After cDNA synthesis, and library preparation, samples were sequenced on DNBSEQ G-400. Sequencing configuration was 28 bp (read1), 8 bp (i7 index), and 91 bp (read2).

### Data analysis

ChIP-seq data were analyzed as follows: the adaptor sequences were trimmed using Trimmomatic^82^, and the sequences were mapped on the honey bee genome assembly Amel_HAv3.1^83^ using Bowtie2^84^. Low-quality reads were eliminated with view -q 20 options using SAMtools^85^. EcR-ChIP peaks were detected with MACS2^25^ using a p-value of 1E−3 as the threshold by comparing them with the data of normal IgG-ChIP. Peaks detected in the mitochondrial chromosome were removed from further analysis. Reproducible peaks were defined as the peaks detected for the 2 biological replicates of each of the foragers and nurse bees (hereafter referred to as forager peaks and nurse bee peaks, respectively). Significance of overlaps between these ChIP-seq peaks were tested by two-side hypergeometric test using all honey bee genes as a population (n = 12,374), which detected the conditional probability of overlapping peaks detected from replicate 2, given the condition that target peaks were detected in replicate 1 out of all honey bee genes using R (ver. 4.0.3). The forager peaks and nurse bee peaks were compared to detect peaks common to both types of workers and peaks unique to each worker type. Overlapping enrichment of common peaks between forager peaks and nurse bee peaks was calculated by a hypergeometric test that detected the conditional probability of the common peaks detected from the target peaks in nurse bee runs, given the condition that forager peaks were detected in forager runs out of all honey bee genes. The candidate EcR target genes were defined as genes closest to the peaks, which we detected using BEDtools^86^. The panels in Supplementary Fig. S2 were drawn using deepTools^87^.

RNA-seq data were analyzed as follows: the trimmed paired end reads were obtained as described above, and mapped onto the honey bee genome with HISAT2^88^. Transcripts count matrices of genes were obtained using StringTie and prepDE.py^89^. Whole output data were normalized and compared using R package TCC-GUI^90^. Differentially expressed genes (DEGs) between foragers and nurse bees, foragers and “BF”, and nurse bees and “BF” were identified at the threshold of p-value < 0.05 and false discovery rate < 0.1.

Single-cell RNA-seq row data were aligned to the honey bee genome using Cell Ranger ver. 5.0.0 and mapped reads with identical cell barcode were retained as RNAs derived from single cell. Downstream analysis was performed using Seurat ver. 4.1.0^91^. Genes expressed in at least 3 cells and cells with at least 200 genes detected were retained for further analysis. After the data were normalized and scaled using a default option, 2000 variable features were found using the “Findvariablefeatures” command for following linear dimension reduction by principal component analysis. Cells were clustered with the parameter of npcs = 40, resolution = 1.3. Gene expression levels for each cluster were averaged and calculated for pseudo-bulk differential expression analysis. Hierarchical clustering was performed using the ward.D2 method. The heatmaps in Supplementary Fig. S4B,C and Fig. 4 were drawn using R package heatmap.2^92^.

### Motif discovery and enrichment analysis

MEME-Suite (https://meme-suite.org/meme/)^93^ was used to find binding motifs of *Am*EcR. First, MEME-ChIP software^94^, which is suitable for motif detection in ChIP-seq peaks, was used for motif discovery. The input DNA sequences comprised 250 bases flanking the peak summits (in total 501 bp) of the ChIP-seq peaks and compared with control sequences randomly extracted from the honey bee genome using the discriminative mode option. Detected candidate motifs were compared with known motifs in the transcription factor binding profiles database JASPAR 2018 (https://jaspar.genereg.net/) using the Tomtom motif comparison tool^95^. CentriMo software was used to compare local preference in each DNA sequence and enrichment of each detected motif with control sequences^96^.

### GO enrichment analysis

*Drosophila melanogaster* orthologs for the honey bee genes were identified using Orthovenn2 (https://orthovenn2.bioinfotoolkits.net/)^97^, g: Orth in g: Profiler (https://biit.cs.ut.ee/gprofiler/)^98^, and protein BLAST in NCBI (https://www.ncbi.nlm.nih.gov/). GO enrichment analysis was conducted using Metascape (https://metascape.org)^99^.

## Supplementary Information

Supplementary Figures.

Supplementary Tables.

## Data Availability

The raw data obtained from RNA-seq, ChIP-seq and single-cell RNA-seq analyses have been deposited on DNA Data Bank of Japan (DDBJ, http://www.ddbj.nig.ac.jp) Sequence Read Archive (DRA) under the accession number DRA014752. The processed files for ChIP-seq, RNA-seq, and scRNA-seq analyses have been deposited on DDBJ Genomic Expression Archive (GEA), and are available under the accession number E-GEAD-565, E-GEAD-566, and E-GEAD-567, respectively.
