# Supplementary material for: Identification of ecdysone receptor target genes in the worker honey bee brains during foraging behavior
Source: Sci Rep. 2023 Jun 28;13:10491. doi: 10.1038/s41598-023-37001-7 (PMC10307900; doi:10.1038/s41598-023-37001-7)
Supplement: Supplementary file 1 — Supplementary Figures. [file 41598_2023_37001_MOESM1_ESM.pdf]

Supplementary Figures

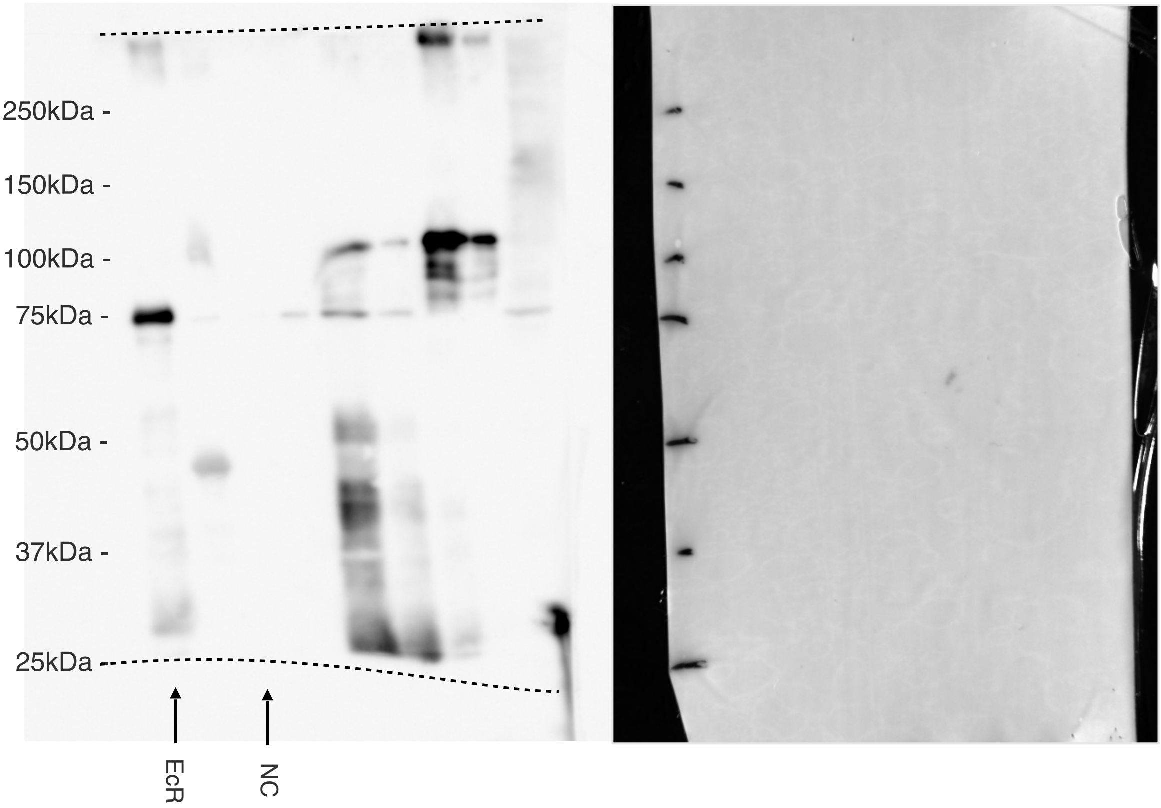

Supplementary Figure S1 Full-length fluorescence and bright field images of the blot of Western blotting using anti-*Am*EcR antibody.

(Left panel) Full-length fluorescence image of the blot for Fig. 1A. Samples are purified *Am*EcR recombinant protein (EcR) and *E. coli* lysate used as a negative control (NC). Arrows indicate the lanes picked out in Fig. 1A. Doted lines indicate the top and bottom edges of the gel. (Right panel) A bright field image of the left panel. The top and bottom edges of the membrane are not visible because they are outside the field of view. Note that molecular weight markers are visible at the leftmost lane of the left panel.

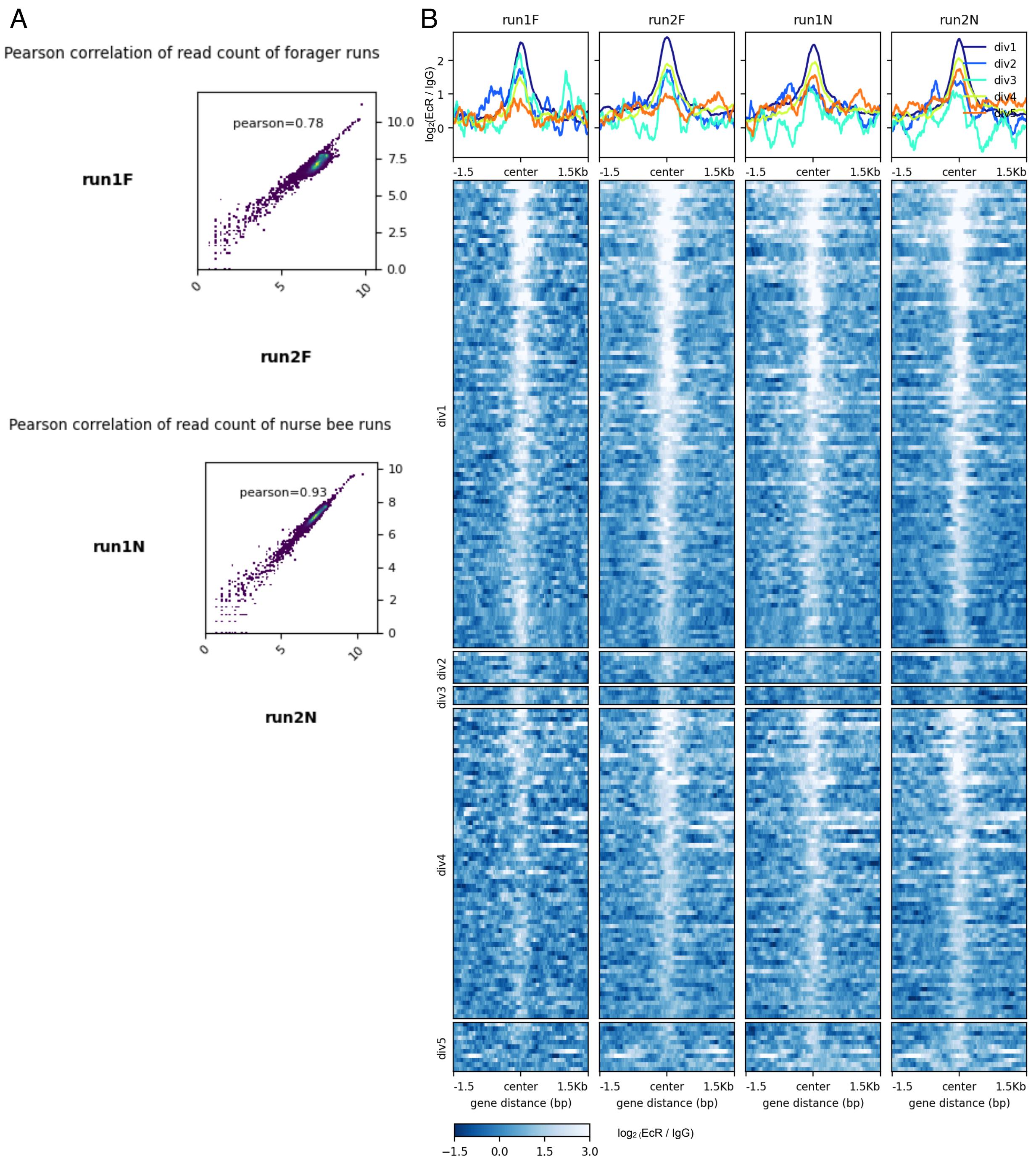

Supplementary Figure S2 Comparison of each ChIP-seq sample.

(A) Pearson correlation plots between both forager runs and both nurse bee runs, respectively. (B)Peak intensity graphs (upper panels) and heat maps (lower panels) of the regions around the peaks. The divisions ("div") 1-5 were classified according to whether the peaks in the regions were called at 1E-3 in each replicate. div1: peaks detected in all runs, div2: peaks detected in two runs of the forager bee and one of the runs of the nurse bee, div3: peaks detected specific to the forager runs, div4: peaks detected in two runs of the nurse bee and one of the runs of the forager, div5: peaks detected specific to the nurse bee runs (lower panels). log_2 fold change was calculated by dividing the read counts in the peak region of EcR-ChIP sample by those of the normal IgG-ChIP sample.

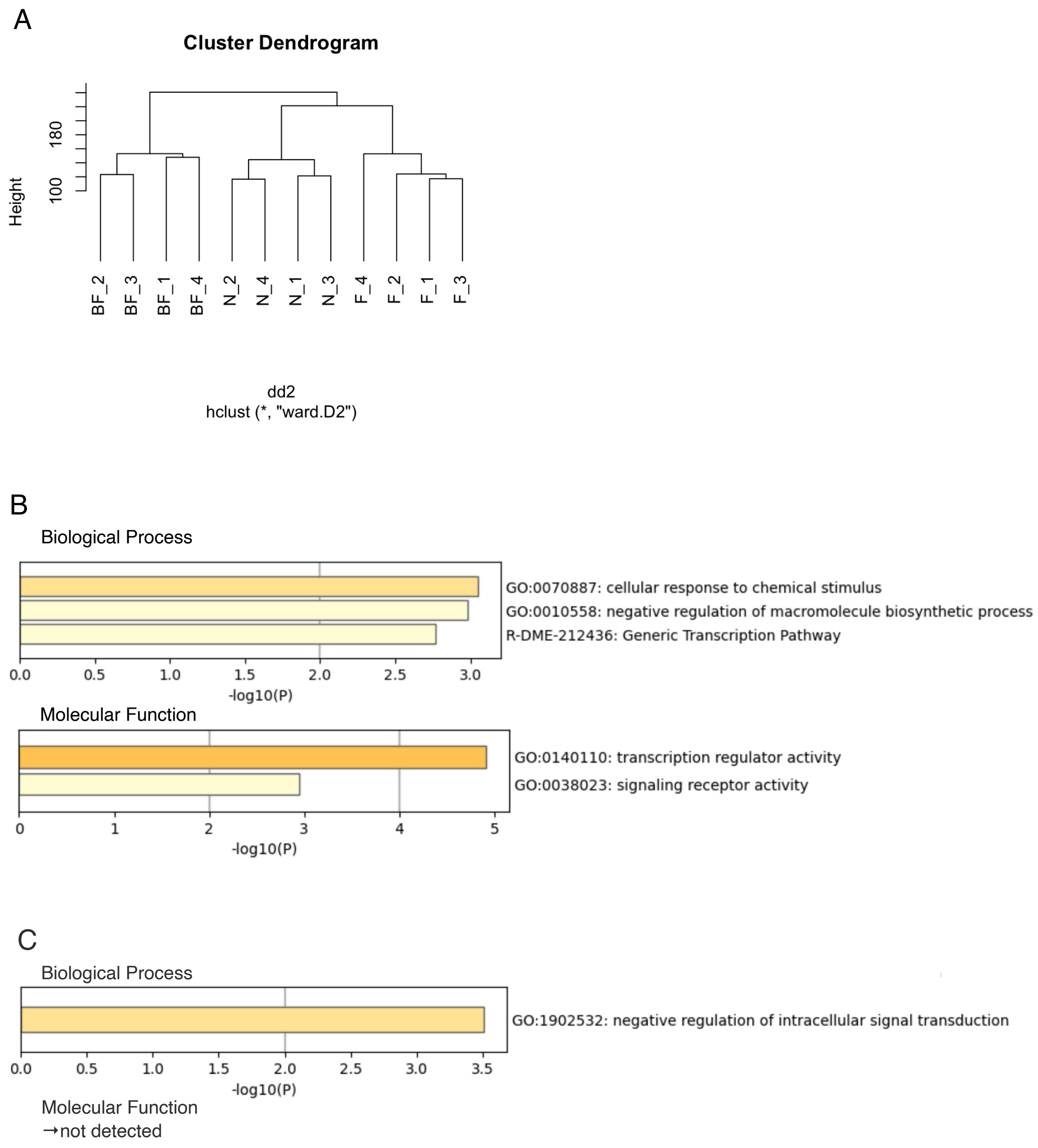

Supplementary Figure S3 Clustering tree and GO enrichment analysis in the RNA-seq analysis.

(A) Clustering tree of RNA-seq samples. GO enrichment analysis of *Am*EcR target genes upregulated during foraging behavior (B) and that of genes expressed differently between BF and F (C) using Metascape.

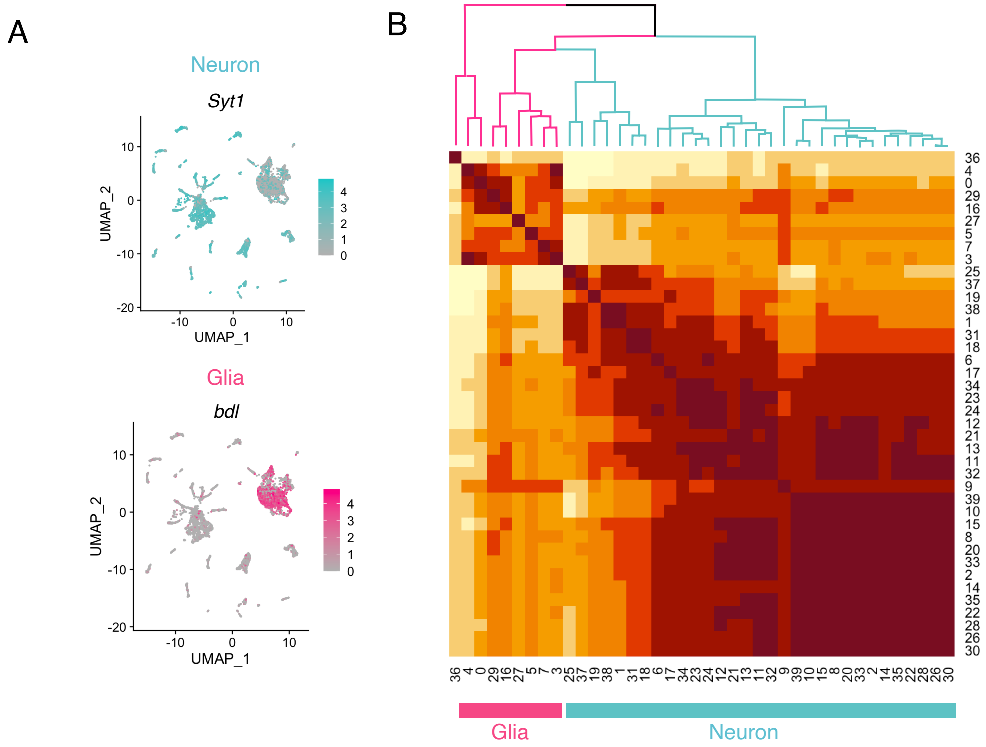

Supplementary Figure S4 Single-cell RNA-seq clustering.

(A) The marker gene expression of glial cells (*bdl*) and neurons (*Syt1*) in the UMAP using Seurat. (B) Correlation plot of each cluster. The clades including glial cells (magenta) and those of neurons (sky blue). (C) Marker gene expression of both projection neurons and glial cells in each cluster.

C

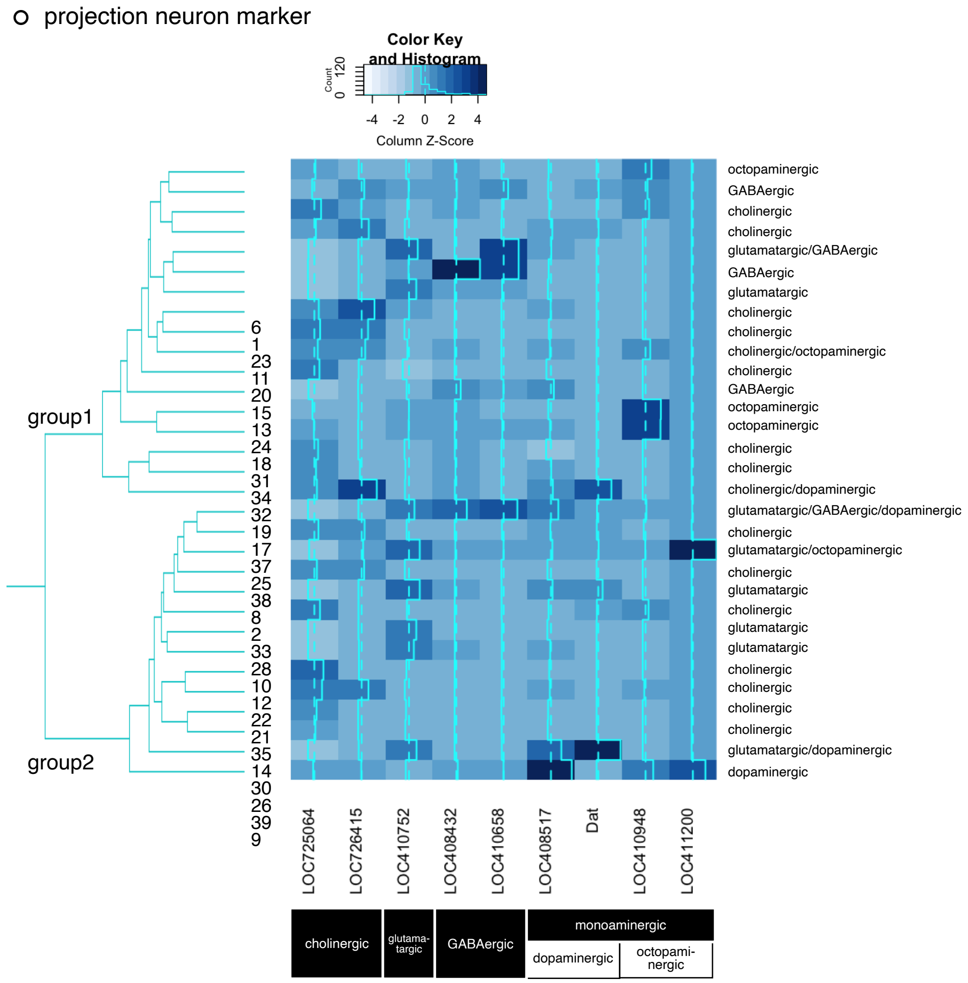

projection neuron marker
Color Key and Histogram
Count
0 120
-4 -2 0 2 4
Column Z-Score
group1
group2
6
1
23
11
20
15
13
24
18
31
34
32
19
17
37
25
38
8
2
33
28
10
12
22
21
35
14
30
26
39
9
octopaminergic
GABAergic
cholinergic
cholinergic
glutamatargic/GABAergic
GABAergic
glutamatargic
cholinergic
cholinergic
cholinergic/octopaminergic
cholinergic
GABAergic
octopaminergic
octopaminergic
cholinergic
cholinergic
cholinergic/dopaminergic
glutamatargic/GABAergic/dopaminergic
cholinergic
glutamatargic/octopaminergic
cholinergic
glutamatargic
cholinergic
glutamatargic
glutamatargic
cholinergic
cholinergic
cholinergic
cholinergic
glutamatargic/dopaminergic
dopaminergic
LOC725064
LOC726415
LOC410752
LOC408432
LOC410658
LOC408517
Dat
LOC410948
LOC411200
cholinergic
glutama-targic
GABAergic
monoaminergic
dopaminergic
octopami-nergic

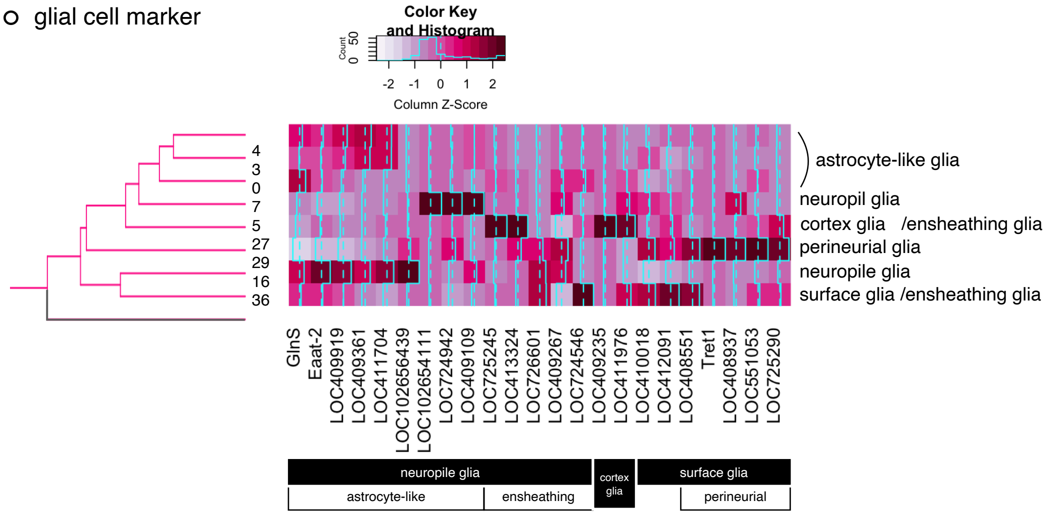

glial cell marker
Color Key and Histogram
Count
0 50
-2 -1 0 1 2
Column Z-Score
4
3
0
7
5
27
29
16
36
astrocyte-like glia
neuropil glia
cortex glia /ensheathing glia
perineurial glia
neuropile glia
surface glia /ensheathing glia
GlnS
Eaat-2
LOC409919
LOC409361
LOC411704
LOC102656439
LOC102654111
LOC724942
LOC409109
LOC725245
LOC413324
LOC726601
LOC409267
LOC724546
LOC409235
LOC411976
LOC410018
LOC412091
LOC408551
Tret1
LOC408937
LOC551053
LOC725290
neuropile glia
astrocyte-like
ensheathing
cortex glia
surface glia
perineurial
